# Honey bee (*Apis mellifera*) gut microbiota promotes host endogenous detoxification capability via regulation of P450 gene expression in the digestive tract

**DOI:** 10.1111/1751-7915.13579

**Published:** 2020-04-27

**Authors:** Yuqi Wu, Yufei Zheng, Yanan Chen, Shuai Wang, Yanping Chen, Fuliang Hu, Huoqing Zheng

**Affiliations:** ^1^ College of Animal Sciences Zhejiang University Hangzhou China; ^2^ USDA‐ARS Bee Research Laboratory Beltsville MD USA

## Abstract

There is growing number of studies demonstrating a close relationship between insect gut microbiota and insecticide resistance. However, the contribution of the honey bee gut microbiota to host detoxification ability has yet to be investigated. In order to address this question, we compared the expression of cytochrome P450s (P450s) genes between gut microbiota deficient (GD) workers and conventional gut community (CV) workers and compared the mortality rates and the pesticide residue levels of GD and CV workers treated with thiacloprid or *tau*‐fluvalinate. Our results showed that gut microbiota promotes the expression of P450 enzymes in the midgut, and the mortality rate and pesticide residue levels of GD workers are significantly higher than those of CV workers. Further comparisons between tetracycline‐treated workers and untreated workers demonstrated that antibiotic‐induced gut dysbiosis leads to attenuated expression of P450s in the midgut. The co‐treatment of antibiotics and pesticides leads to reduced survival rate and a significantly higher amount of pesticide residues in honey bees. Taken together, our results demonstrated that honey bee gut symbiont could contribute to bee health through the modification of the host xenobiotics detoxification pathways and revealed a potential negative impact of antibiotics to honey bee detoxification ability and health.

## Graphical abstract

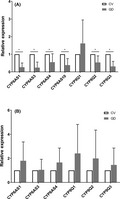

## Introduction

The honey bee (*Apis* spp.) is one of the most important insect pollinators of monoculture food crops worldwide, adding over $15 billion in economic value to agriculture each year in the United States and more than 200 billion worldwide (Klein *et al.*, 2007; Gallai *et al.*, 2009; Meixner, 2010). However, global honey bee populations have undergone elevated colony losses during the last decade (Zee *et al.*, 2014; Brodschneider *et al.*, 2016; Brodschneider *et al.*, 2018). Honey bees are exposed to a wide range of pesticides while foraging in the field or consuming contaminated food stocked in the hive, such as the widely used neonicotinoid insecticides. Although direct mortality from pesticide is considered to be limited in the field environment, sublethal doses of pesticides have recently been found to affect honey bee behaviour, foraging ability, learning, individual and colony development (Gill *et al.*, 2012; Henry *et al.*, 2012; Fairbrother *et al.*, 2014). In addition, synergistic interactions between pesticides and other stressors including pathogens and parasites have also been identified and reported (Doublet *et al.*, 2015; Goulson *et al.*, 2015). Therefore, pesticides are considered as one of the possible stressors causing honey bee colony losses and the general decline of the honey bees (Goulson *et al.*, 2015). Furthermore, bees also encounter certain chemicals that are used in beehive, like acaricides for mite control (Johnson, 2015) and antibiotics for American or European foulbrood diseases (Mutinelli, 1996).

Metabolic detoxification is a major mechanism accounting for insect resistance to xenobiotic toxins. Cytochrome P450 monooxygenases (CYPs, also called P450s), carboxylesterases (COEs) and glutathioneS‐transferases (GSTs) are the three major insect detoxification enzyme systems (Berenbaum and Johnson, 2015). Studies have demonstrated the involvement of honey bee P450 enzymes in the detoxification of pesticides and secondary metabolites from plants. For example, P450 inhibitor piperonyl butoxide (PBO) was found to enhance the toxicity of pyrethroid insecticides and neonicotinoid insecticides on honey bees (Iwasa *et al.*, 2004; Johnson *et al.*, 2006). Mao *et al.* (2017) discovered that myclobutanil inhibits the P450‐mediated detoxification of dietary phytochemicals, which leads to the interference of ATP production and insufficiency of energy for flight and other activities. CYP6AS1, CYP6AS3, CYP6SA4 and CYP6AS10 of the CYP6 subfamily are found to be involved in the metabolism of quercetin, a flavonoid component of honey and pollen (Mao *et al.*, 2009), and CYP9Q1, CYP9Q2 and CYP9Q3 are found to play an important role in the metabolism of *tau*‐fluvalinate, an acaricide used for mite control in honey bee colonies (Mao *et al.*, 2011). In addition, functional expression of the entire CYP3 clade of honey bee P450s identified that CYP9Q3 also metabolizes thiacloprid with high efficiency and transgenic *Drosophila* lines expressing honey bee CYP9Q3 showed marked and significant resistance to thiacloprid compared with control flies of the same genetic background (Manjon *et al.*, 2018). These studies have demonstrated that P450 monooxygenases contribute significantly to honey bee xenobiotic detoxification and tolerance of pesticides.

The complex microorganisms that populate the animal gastrointestinal tract are emerging as key players in governing host health and disease. In the past few years, researches have demonstrated an association between symbiotic microbes and insecticide resistance in insects (Pietri and Liang, 2018). Arismendi *et al. *(2015) found that the colonization of *Candidatus* Phytoplasmaulmiin *Amplicephalus curtulus* Linnavuori & DeLong leafhoppers leads to an increased β‐esterase and GST activity. Likewise, midgut symbiotic bacteria can boost the activity of several enzymes involved in xenobiotic metabolism in the mosquito *Anopheles stephensi* (Soltani *et al.*, 2017). These studies demonstrated a potential that insect gut symbionts could modify host insecticide resistance through the regulation of host endogenous detoxification pathways.

In recent years, a growing number of studies have demonstrated a close link between the gut microbiome and the health of honey bees. The bee gut is colonized by eight core bacterial phylotypes, including *Snodgrassella alvi*, *Gilliamella apicola*, *Lactobacillus* spp, *Bifidobacterium* spp, *Frischella perrara*, *Bartonella apis*, *Parasaccharibacter apium* and *Commensalibacter* spp (Kwong *et al.*, 2017). Similar to that of other insects, honey bee gut microbiota participates in host food digestion, promotes host weight gain via bacterial metabolism and hormonal signalling, and plays roles in protection against pathogen infection as well as in regulation of the host immunity system (Kwong and Moran, 2016; Raymann and Moran, 2018). In addition, Schwarz *et al. *(2016) showed that honey bee gut dysbiosis leads to the change of P450 gene expression. However, our current knowledge of the involvement of honey bee gut microbiota in detoxification is still very limited. Hence, the current study aimed to investigate the interaction between honey bee (*Apis mellifera*) gut microbiota, honey bee resistance to pesticides and the honey bee endogenous detoxification system, with a focus on the well‐researched honey bee P450 detoxification enzymes. For the first time, we showed that honey bee gut microbiota promoted the expression of P450 detoxification enzymes in the midgut. And, the P450 expression induced by bacterial colonization contributed to the increase of survivorship of bees treated with a sublethal dose of thiacloprid or fluvalinate. These findings suggested that honey bee gut microbiota is closely linked with host xenobiotic detoxification capability, thereby enhancing host resistance to pesticides. In addition, recent studies have demonstrated that antibiotics may damage the beneficial bacteria in the guts of honey bees and make them more prone to deadly infections (Li *et al.*, 2017; Raymann *et al.*, 2017). In this study, we found that antibiotic treatment significantly attenuated the expression of certain key detox honey bee P450s in the midgut, and that co‐treatment of antibiotics with thiacloprid and fluvalinate led to reduced survivorship and increased pesticide residues within the honey bee, suggesting that dysbiosis resulting from antibiotic exposure affects honey bee detoxification capability and honey bee health.

## Results

### Establishment and validation of experimental worker bee models

We prepared gut microbiota deficient (GD) workers and conventional gut community (CV) workers to characterize the effects of gut microbiota on the host detoxification ability. Newly emerged germ‐free workers were either kept microbiota depleted or colonized with gut homogenates for 5 days to allow for the establishment of a normal gut microbiota community. This procedure resulted in a total bacterial load of around 10^5^ cells per gut of GD bees and a total of approximately 10^10^ bacterial cells per gut of CV bees (Fig. S1). Antibiotic treated (AT) workers and normally fed (NF) workers were prepared to characterize the effects of gut microbiota dysbiosis on the detoxification ability of the honey bees. Newly emerged workers were colonized with the gut homogenates for 5 days and AT bees were then treated with 400 μg ml^−1^ of tetracycline, which was shown to perturb the bee gut microbiota (Raymann *et al.*, 2017), for another 5 days. Due to the possibility that measurements based on bacterial DNA were partly obscured by DNA from dead bacterial cells, RNA samples were used to determine the bacterial loads in AT and NF workers (Motta *et al.*, 2018). The expression level of bacteria 16S rRNA in NF workers was significantly higher than that in AT workers at day five post‐antibiotic treatment (Fig. S1). These analyses have validated the successful establishment of the gut microbiota deficient worker model and gut microbiota dysbiosis worker model that can be used for downstream experiments.

### Gut microbiota enhances the P450 expression in the honey bee midgut

To evaluate the impact of gut microbiota depletion on honey bee detoxification pathways, the expression of seven P450 genes (*CYP6AS1*, *CYP6AS3*, *CYP6AS4*, *CYP6AS10*, *CYP9Q1*, *CYP9Q2* and *CYP9Q3*, whose roles in honey bee xenobiotic detoxification have been confirmed by heterologous expression experiments) in the midgut and hindgut of GD and CV workers were analyzed and compared.

**Fig. 1:**
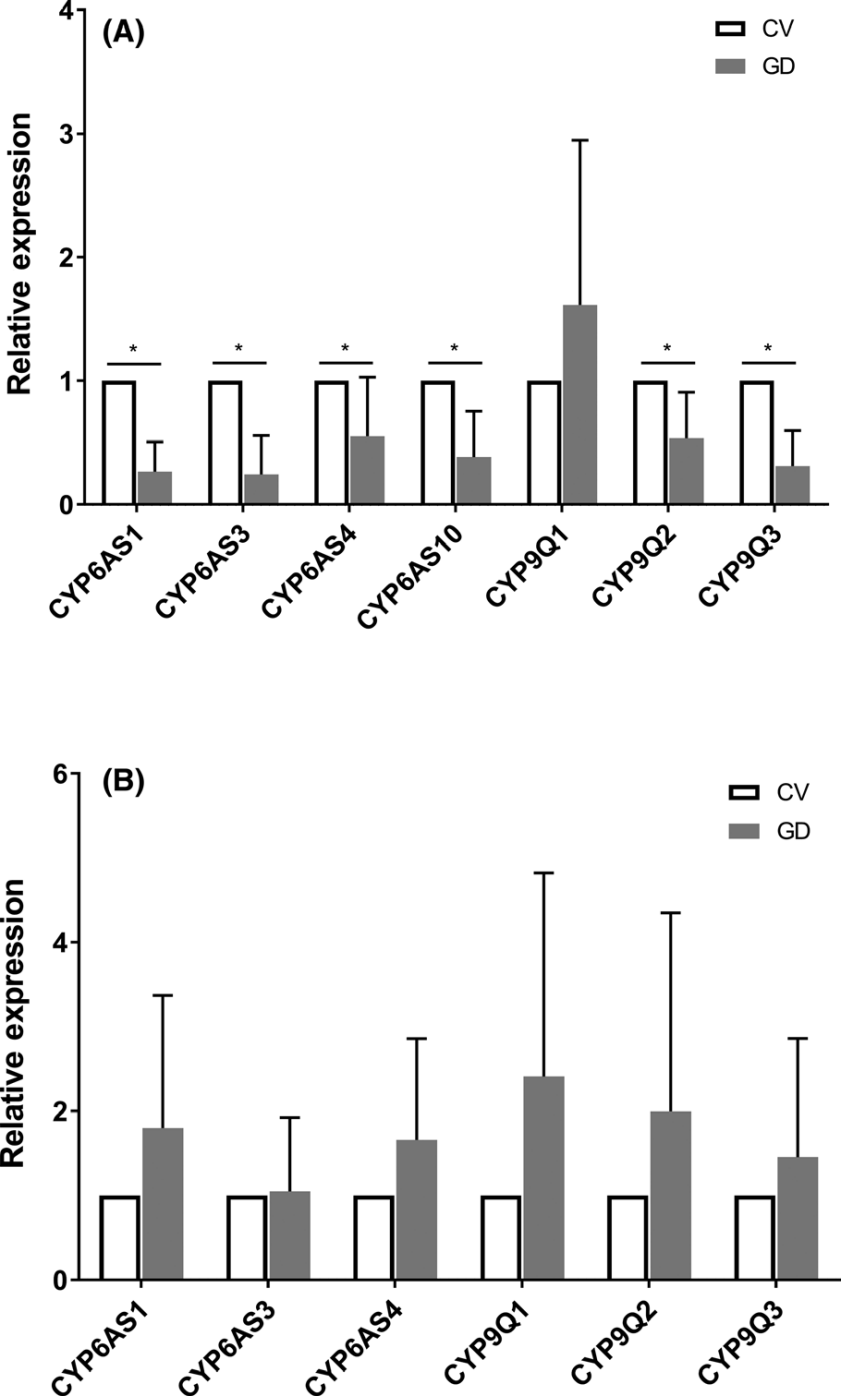
Differential expression of cytochrome P450s in midgut (A) or hindgut (B) of GD and CV workers (*n* = 6 colonies). Error bars represent SD fold changes. ‘*’ represents significant difference (*P* < 0.05, independent sample *t*‐test). CV, conventional gut community workers; GD, gut microbiota deficient workers.

Compared to GD workers, the expressions of *CYP6AS1*, *CYP6AS3*, *CYP6AS4*, *CYP6AS10*, *CYP9Q2* and *CYP9Q3* were significantly upregulated in the midgut of CV bees (*P* < 0.05), while the expression of *CYP9Q1* was not altered (Fig. 1A). In contrast, the expression of all of these P450 genes in the hindgut was not significantly affected by the colonization of gut microbiota (Fig. 1B) except for *CYP6AS10*, which was found to be barely detectable in the hindgut in this study (*C*_q_ > 35 cycles).

### Gut microbiota deficiency negatively affects the viability and pesticide metabolism activity of pesticide‐treated workers

**Fig. 2:**
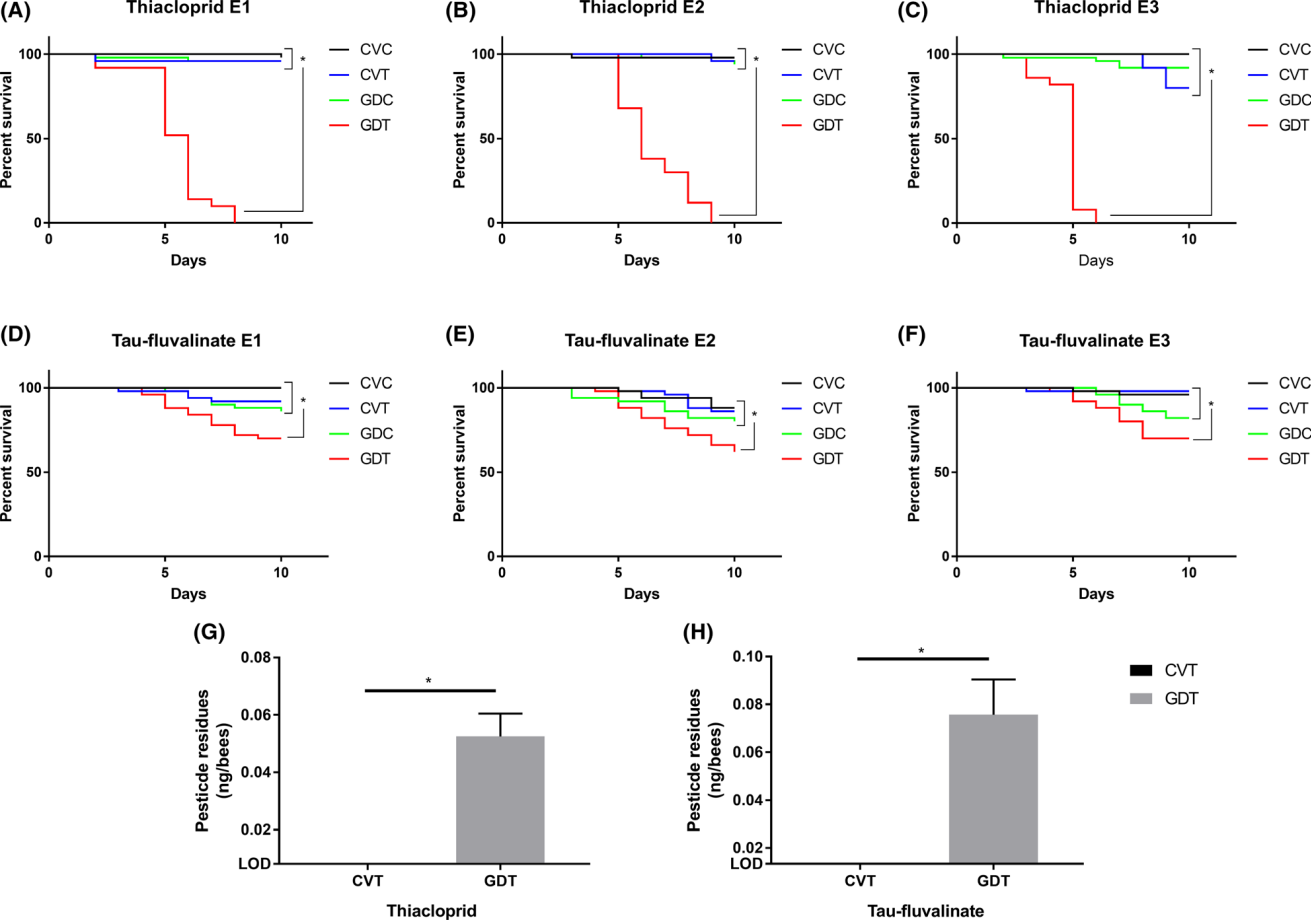
Changes of honey bee susceptibility and metabolism to pesticides. A–F. The percent survival of workers after thiacloprid or tau‐fluvalinate exposure, shown as a Kaplan–Meier survival curve. * represents significantly different between comparison (*P* < 0.05, log‐rank test). E1, E2 and E3 represent three replicates using different colony. G. Comparison of thiacloprid (left) and tau‐fluvalinate (right) levels between dissected (abdomen excised) GD and CV bees (*n* = 30 bees × 3 replicate cages). “*” represents significant difference (*P* < 0.05, one‐way ANOVA). CVC, conventional gut community workers treated with DMSO; CVT, conventional gut community workers treated with pesticide, GDC, gut microbiota deficient workers treated with DMSO; GDT, gut microbiota deficient workers treated with pesticide.

We investigated if the GD workers and CV workers would react differently to pesticides exposure. GD workers and CV workers were treated with a sublethal dose of thiacloprid (35 mg l^−1^ in syrup) or fluvalinate (400 mg l^−1^ in syrup) for 10 days; hereafter, these were referred to as GD workers treated with pesticide (GDT) and CV pesticide treated workers (CVT). GD workers and CV workers treated with dimethyl sulphoxide (DMSO) solvent were referred to as GD control workers (GDC) and CV control workers (CVC). In all three replicates of thiacloprid and fluvalinate exposure experiments, the survival rate of GDT workers was significantly lower when compared to that of GDC, CVT or CVC workers (Fig. 2A–F). The mortality rate of GDT workers reached 100% and 40% after 10 days of chronic exposure to thiacloprid or fluvalinate, respectively. Meanwhile, no significant difference was observed among the survivorship of GDC, CVT and CVC workers (Fig. 2A–F), suggesting that just removing gut microbiota or treating workers with sublethal doses of pesticides had no obvious negative effect on the lifespan of workers during the experimental period.

To determine whether these increased mortalities were attributable to the changes of pesticide metabolism caused byP450 downregulation, we measured the pesticide residues in the honey bees’ body system using HPLC. As shown in Fig. 2G, both thiacloprid and fluvalinate levels were significantly higher in GDT workers than in CVT workers after 4 days and 5 days after exposure to thiacloprid and fluvalinate, respectively. And no thiacloprid or fluvalinate was detected in CVC and GDC samples (data not shown).

### Thiacloprid and fluvalinate are not metabolized by whole bee gut cultures in vitro

In order to investigate the possibility that honey bee gut microbiota can metabolize thiacloprid or fluvalinate, we performed *in vitro* experiments in which entire hindgut homogenates were isolated from CV or GD bees and cultured in the presence of thiacloprid and fluvalinate. The supernatants of the bacterial cultures were extracted and analysed using HPLC. No significant changes were found in CV gut homogenates when compared to GD gut homogenates after 2 days of culture (Fig. S2).

### PBO treatment increased the mortality of pesticide treated workers

**Fig. 3:**
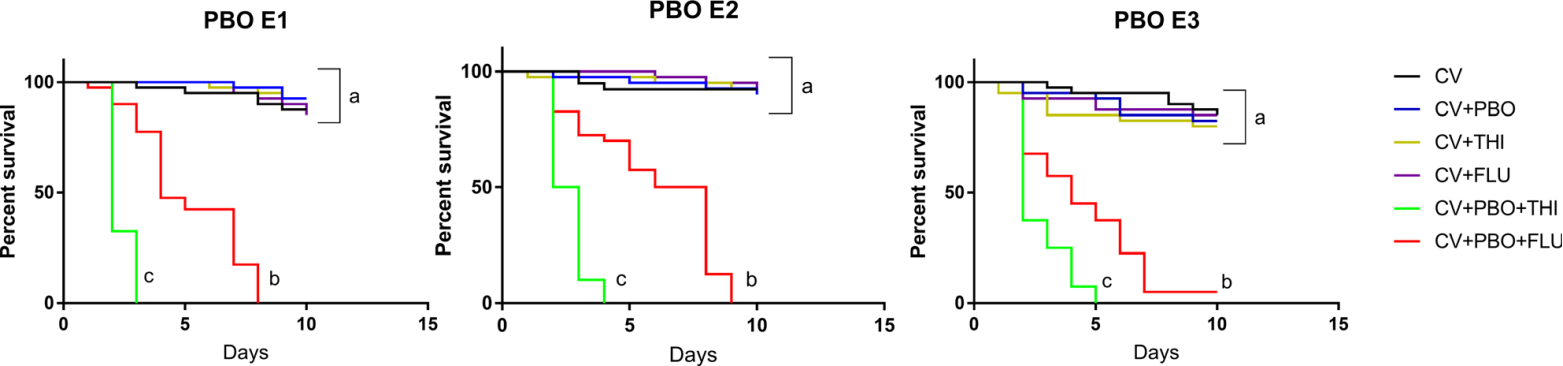
The percent survival of workers after co‐treatment of PBO with thiacloprid or fluvalinate exposure, shown as a Kaplan–Meier survival curve. Different letters represent significantly different between comparison (*P* < 0.05, log‐rank test). E1, E2 and E3 represent three replicates using different colony. CV, conventional gut community workers; CV + PBO, conventional gut community workers treated with PBO; CV + THI, conventional gut community workers treated with thiacloprid; CV + FLU, conventional gut community workers treated with fluvalinate; CV + PBO+THI, conventional gut community workers treated with both PBO and thiacloprid; CV + PBO+FLU, conventional gut community workers treated with both PBO and fluvalinate.

To confirm that P450s are involved in the honey bee resistance to thiacloprid and fluvalinate, CV workers were co‐treated with 0.1% PBO, a widely used P450 enzyme inhibitor (Johnson *et al.*, 2006), and thiacloprid (35 mg l^−1^ in syrup) or fluvalinate (400 mg l^−1^ in syrup). In all three replicates, we found that PBO treatment significantly increased the mortality of thiacloprid or fluvalinate treated workers (Fig. 3).

### The disruption of honey bee gut bacteria by antibiotic negatively impacts the expression ofP450, the lifespan and pesticide metabolism of pesticide‐treated workers

**Fig. 4:**
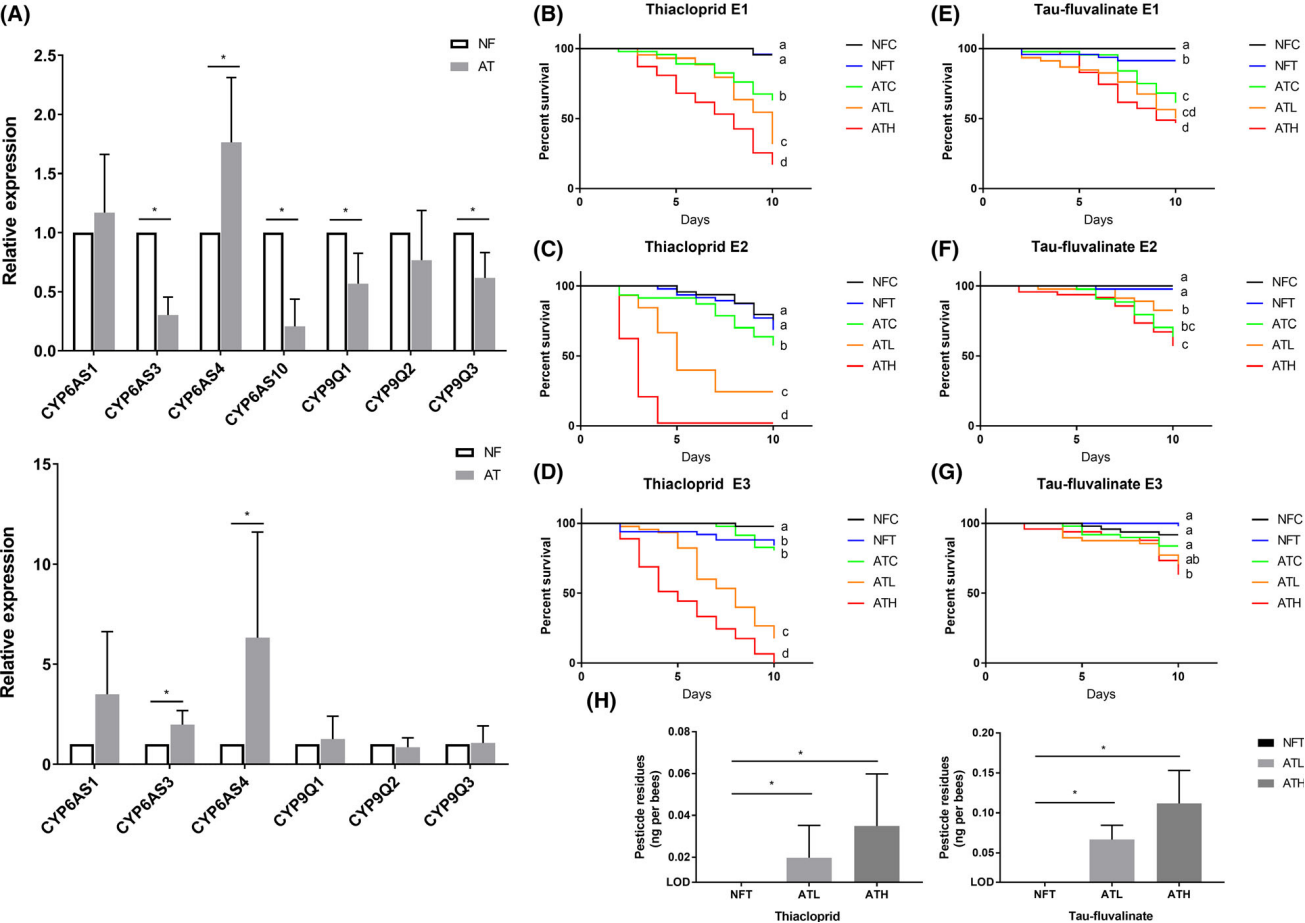
Impacts of antibiotic exposure on honey bee P450 expression, metabolism and susceptibility to pesticides. A. changes of cytochrome P450 expression s in midgut (upper part of the panel) and hindgut (lower part of the panel) of AT and NF workers (*n* = 6 colonies). Error bars represent SD fold changes. ‘*’ represents significant difference (*P* < 0.05, independent sample *t*‐test). B–G. The percent survival of workers after thiacloprid or fluvalinate exposure, shown as a Kaplan–Meier survival curve. Different letters represent significantly different between comparison (*P* < 0.05, log‐rank test). E1, E2 and E3 represent three replicates using different colony. H. Comparison of thiacloprid (left) and tau‐fluvalinate (right) levels between dissected (abdomen excised) AT and NF bees (*n* = 30 bees × 3 replicate cages). ‘*’ represents significant difference (*P* < 0.05, one‐way ANOVA). ATC, antibiotic treated workers treated with DMSO; ATH, antibiotic treated workers treated with high dosage pesticide; ATL, antibiotic treated workers treated with low dosage pesticide; NFC, normally fed workers treated with DMSO, NFT, normally fed workers treated with pesticide.

Similar to the gut microbiota depletion, gut microbiota dysbiosis caused by antibiotic also changed the P450 gene expressions in the midgut. Of the seven P450 genes, expression of *CYP6AS3*, *CYP6AS10*, *CYP9Q1* and *CYP9Q3* was suppressed while *CYP6AS4* was induced in the midgut of AT workers (Fig. 4A). In the hindgut, we found that the expression of *CYP6AS3* and *CYP6AS4* was upregulated by the dysbiosis (Fig. 4A).

Then, we measured the susceptibility of tetracycline‐treated workers to pesticides. According to their treatments, AT workers and NF workers were referred to as NFC (NF workers treated with DMSO), NFT (NF workers treated with 35 mg l^−1^ thiacloprid or 400 mg l^−1^ fluvalinate in syrup), ATC (AT workers treated with DMSO), ATL (AT workers treated with 17.5 mg l^−1^ thiacloprid or 200 mg l^−1^ fluvalinate in syrup) and ATH (AT workers treated with 35 mg l^−1^ thiacloprid or 400 mg l^−1^ fluvalinate in syrup) workers. In all the replicates, microbial imbalanced workers exhibited increased susceptibility to thiacloprid. The survival rates of ATL and ATH workers were significantly lower than that of NFC or NFT workers. Furthermore, both ATL and ATH workers decreased in survivorship when compared to ATC workers (Fig. 4B–D). As for fluvalinate treatment, significant difference on the mortality between ATH and ATC was observed in replicates 1 and 3, however, the survival rates of ATL workers were not strongly affected by fluvalinate (Fig. 4E–G). In addition, significant differences were observed between the mortality rate of NFC workers and ATC workers (except for one replicate), which is in accordance with previous research (Raymann *et al.*, 2017) and confirmed that imbalanced gut microbiota itself is also harmful to honey bees.

According to their survival rates, workers treated with thiacloprid were sampled for pesticide residue analysis on day four post‐treatment and workers treated with fluvalinate were sampled on day seven post‐treatment. Thiacloprid and fluvalinate levels were significantly higher in ATL workers and ATH workers compared with NFT workers, for whom the pesticide residues were below the limit of quantitation (LOQ). However, no difference was found between ATL and ATH workers (Fig. 4H).

### Gut microbiota has no impact on the expression of detoxification P450s in antenna or legs

In addition to the expressions in intestinal segments, P450s function in detoxification are also expressed in anatomical structures associated with regular contact with xenobiotics, such as legs and antennae. We therefore analysed the expressions of our target P450s in the legs and antennas of GD and CV workers. Our results showed that none of these genes were significantly altered in these tissues (Fig. S3); this demonstrated that the gut microbiota may only influence the P450 expression in the honey bee digestive tract and that expression of P450 genes in other honey bee tissues is not co‐regulated.

## Discussion

Cytochrome P450 enzymes are the major contributors to honey bee detoxification (Berenbaum and Johnson, 2015). In the current study, we focused on the interaction of honey bee gut microbiota and honey bee endogenous detoxification enzyme. Given the important role that honey bee P450 monooxygenase enzymes play in detoxification, our study provides important insights into the functional roles of gut bacteria as well as the interactions between gut microbiota and host detoxification capability in the honey bee.

The midgut is one of the main sites for detoxification in insects (Smagghe and Tirry, 2001). Pesticides can be taken up by the midgutepithelial cells, where most of it is metabolized before being transported back into the midgut lumen across the apical membrane or into the haemolymph across the basal membrane (Esther *et al.*, 2017). Our results showed that gut microbiota strongly promotes the expression of key enzymes of the honey bee xenobiotic detoxification pathway. Six of the seven honey bee P450 detoxifying enzymes were upregulated in the midgut of CV workers, suggesting that honey bee gut microbiota enhance host detoxification capability and manipulate host metabolism. This is in accordance with related researches on mammals that demonstrated the importance of microbial activity in metabolic phenotype development. Toda et al. (Toda *et al.*, 2009) reported that most of the major CYP isozymes were highly expressed in the livers of specific‐pathogen‐free mice compared with germ‐free mice. Claus et al. (Claus *et al.*, 2011) found that microbiota stimulates the expression and activity of major hepatic drug‐metabolizing P450s. In the meanwhile, it is quite interesting to note that the P450 expressions in the hindgut were not influenced, though most of the honey bee core gut bacteria are colonized in the hindgut instead of the midgut (Martinson *et al.*, 2012). The results that gut microbiota only influenced the P450 expression in honey bee midguts suggests that honey bee gut microbiota may have a different effect on the different parts of the gastrointestinal tract, which may be correlated with the bacterial abundance and composition differences (Martinson *et al.*, 2012) or the physiological differences among different compartments of the bee gut. Therefore, further studies are needed to determine which cellular mechanisms underlie the observed regulatory function of gut microbiota and to explain why the P450 expressions are only influenced in the midgut. Collectively, our findings on the expression change of P450s indicated that gut microbiota has a strong positive effect on honey bee detoxification enzyme expression, which are vital to the detoxification ability and insecticide resistance of honey bees.

Neonicotinoid insecticides are an important group of neurotoxins specifically acting as antagonists of the insect nicotinic acetylcholine receptors (Matsuda *et al.*, 2001). Currently, neonicotinoid insecticides are considered as one of the main threats to honey bee health. Many lethal and sublethal effects of neonicotinoid insecticides on bees have been described in laboratory and field studies over the past decades (Blacquiere *et al.*, 2012; Rundlöf *et al.*, 2015; Tsvetkov *et al.*, 2017). Of all the widely used neonicotinoid insecticides, thiacloprid has relatively low toxicity to honey bees (Iwasa *et al.*, 2004), due to the fact that CYP9Q3 can metabolize thiacloprid with high efficiency (Manjon *et al.*, 2018). Pyrethroids exert their toxic effects by disrupting the function of voltage‐gated sodium channels which are critical for electrical signalling in the nervous system (Soderlund and Bloomquist, 1989). *Tau*‐fluvalinate, a typical pyrethroid pesticide, is widely used in honey beehives as an acaricide for the control of devastating*Varroa*mites. The long‐term application of fluvalinate as an apicultural tool as well as its absorption by the wax in the hive have resulted in a high‐level of fluvalinate residue in bee colonies all over the world (Johnson *et al.*, 2010). Fluvalinate is considered harmless to bees under normal circumstances (Johnson *et al.*, 2006), because members of the honey bee CYP9Q subfamily, namely CYP9Q1, CYP9Q2 and CYP9Q3can efficiently metabolize fluvalinate (Mao *et al.*, 2011). As expected, GDT bees administered with thiacloprid displayed a dramatically increased mortality rate and a higher level of thiacloprid residues compared with CVT workers. We also found that the innoxious fluvalinate became fatal when applied to GD workers, and the fluvalinate remaining in GDT workers was significantly higher than in CVT workers. Clearly, these findings showed that honey bee gut bacteria influence the metabolism of pesticide and confirmed that gut microbiota is crucial to honey bees for their pesticide tolerance.

Still, there are plenty of studies that showed that insect bacteria have the ability to metabolize pesticides directly (Cheng *et al.*, 2017; Dada *et al.*, 2019), so we conducted an *in vitro* experiment to examine the possibility that the gut microbiota directly detoxifies the chemical pesticides and leads to resistance. Our results showed that neither of these two pesticides were significantly degraded by honey bee whole gut cultures *in vitro*, suggesting that the resident honey bee gut bacteria are not likely to degrade these two pesticides. However, future works using isolated bacteria strains are needed to provide a better understanding of the direct detoxification ability of honey bee gut symbionts. Then we have co‐treated CV workers with both PBO and pesticide, we found that PBO treatment significantly reduced the honey bee survival rate, which is in accordance with previous studies (Iwasa *et al.*, 2004; Johnson *et al.*, 2006) and provided direct evidence for the involvement of P450 enzymes in thiacloprid and fluvalinate detoxification in the presence of the microbiota. Taken together, our results revealed that honey bee gut microbiota enhances host resistance to thiacloprid and fluvalinate through the regulation of the host endogenous detoxification mechanism, instead of direct degradation of toxins by gut symbiont. In addition, considering that P450 enzymes are capable of oxidizing many different substrates (Munro *et al.*, 2013), we believe that the contribution of gut microbiota enhanced P450 expression to honey bee pesticide resistance is not limited to these two pesticides investigated in our study.

Antibiotics have been a cornerstone of innovation in the fields of public health, agriculture and medicine. However, recent studies have shed new light on the collateral damage they impart on the indigenous host‐associated communities (Modi *et al.*, 2014). Zhan et al. (Zhan *et al.*, 2018) revealed that the oral bioavailability of triazine herbicides was significantly increased in the rats treated with ampicillin or antibiotic cocktails, which is a consequence of the alteration of hepatic metabolic enzyme gene expression and intestinal absorption‐related proteome. In apiculture, antibiotics are frequently used in bee colonies to prevent bacterial infection. Recent studies have demonstrated that antibiotic exposure can disrupt both the size and composition of the honey bee gut microbiome (Raymann *et al.*, 2017; Raymann *et al.*, 2018a), resulting in impaired metabolism, weakened immunity and decreased survivorship (Li *et al.*, 2017; Raymann *et al.*, 2017; Li *et al.*, 2019). In our study, tetracycline, a commonly used antibiotic in bee keeping, was employed in field doses to workers. We observed a significantly decreased the community size after 5 days after antibiotic treatment and a decrease in survival rate, similar to previous studies (Li *et al.*, 2017; Raymann *et al.*, 2017). These confirmed a successful establishment of a gut dysbiosis worker model and once again proved the detrimental effect of antibiotic on honey bee longevity.

In light of our findings above, we further evaluated whether the gut microbiota dysbiosis caused by antibiotic has a negative impact on honey bee detoxification ability, which might be a problem we will encounter in beekeeping. As predicted, our results displayed that gut microbiota dysbiosis downregulated the expression of P450s in the midgut, therefore, attenuating the honey bees’ detoxification ability. Interestingly, the expression changes of P450 in the midgut caused by the gut microbiota dysbiosis are quite different from gut microbiota deficiency, and the expression of two P450s functioning to metabolize phytochemicals (*CYP6AS3* and *CYP6AS4*) was induced in the hindgut of AT workers. This may be due to the difference of metabolites in the gut of AT and of GD workers and suggests that honey bee gut microbiota deficiency and dysbiosis have different impacts on host physiology. The administration of both thiacloprid and fluvalinate on AT workers led to significantly increased mortality compared with that of pesticide treated NF workers. The pesticide remaining in the AT workers was significantly increased, which was probably caused by the downregulation of P450s in the midgut. These results demonstrated that the application of antibiotics interrupts the P450 expression in honey bee digestive tracts and enhances the pesticide risks for honey bees, even those of low toxicity to honey bees. The doses of pesticides we applied in this study were higher than actual field levels (38), suggesting that the combination of antibiotics and pesticides might not lead to an acute death of workers in the field colonies. Still, it is possible to hypothesize that gut dysbiosis could enhance the sublethal effects of pesticides, especially during the overwintering period when workers are exposed to antibiotics and pesticides (43) for a long period of time, and eventually lead to colony loss. However, our experiments were carried out using caged bees in a laboratory environment only, where workers have no route for acquisition of the gut microbiota and normally do not defecate. Thus, the combinatory effects of antibiotics and pesticides in field colonies remain to be determined. Moreover, it is worth studying the impact on honey bee detoxification of other chemicals (Kakumanu *et al.*, 2016; Motta *et al.*, 2018; Nogrado *et al.*, 2019), that also perturb the gut microbial balance in honey bees.

## Conclusion

Here in this study, our work revealed the interaction between honey bee gut microbiota and host resistance to pesticides for the first time. Our results showed that honey bee gut microbiota promotes the expression of detoxification enzymes in the midgut, which contribute to the host endogenous detoxification and resistance to thiacloprid and fluvalinate. These findings proved a close relationship between gut microbiota and honey bee detoxification capability, provided new insights into the honey bee host‐microbiome interaction and perspectives for future studies on host‐gut microbial metabolic interaction. In the current study, we have demonstrated a synergistic interaction between antibiotics and pesticides, which is detrimental for bees. And our results suggested this may be due to the reduced detoxification ability of honey bee. We were able to point out the beneficial role of a balanced gut microbiome in honey bees and provide fundamental information on how antibiotic treatment affects honey bee health.

## Experimental procedures

### Rearing of honey bees

Honey bee (*Apis mellifera*) colonies were kept in an apiary maintained at the Honey Bee Research Laboratory in the College of Animal Sciences, Zhejiang University, Hangzhou, China. All of the workers reared in the laboratory were kept in bee‐rearing cages and incubated at 31 ± 1°C and 75 ± 5% relative humidity (RH). The number of dead bees was recorded daily and removed. All pollen supplied to workers were irradiation sterilized.

GD workers and CV workers were obtained using the protocol described by Zheng *et al.* (2017). Briefly, late‐stage pupae (dark‐eyed) were removed from brood frames and transferred to sterile dishes. The dishes were placed in an incubator at 34 ± 1°C with 80 ± 5% RH until bees emerged. Workers emerged between 24 and 48 h after transformation were collected for experiments, then newly emerged germ‐free bees were randomly assigned to GD or CV groups (40 workers per cage). GD workers were supplied with pollen and sterile sugar water (50% sucrose solution, w/v), meanwhile, CV workers were supplied with food containing homogenates of freshly dissected guts of workers from their original hives for 5 days and then switched to pollen and sterile sugar water. The experiment was replicated using six different colonies.

AT workers and NF workers were obtained using a protocol described by Raymann *et al.* (2017) with slight modifications. In brief, sealed brood combs containing emerging adult workers were removed from a colony and placed in an incubator at 34 ± 1°C with 80 ± 5% humidity overnight. The following day, newly emerged workers were randomly assigned to AT or NF groups (50 per cage). In each cage, workers were supplied with food containing homogenates of freshly dissected guts of workers for 5 days, then AT workers were treated with 400 µg ml^−1^ of tetracycline suspended in sterile sugar water while NF workers were fed sterilized sugar water. The experiment was replicated using six different colonies.

### Sampling of workers for qPCR

Five GD and CV workers from each cage were sampled on day five after emergence and their guts were immediately dissected. Then, each sampled gut was immediately placed in chilled vials and used for DNA extraction. DNA was extracted from the gut using TIANamp Stool DNA Kit (Tiangen Biotech Co., Ltd, Beijing, China) according to the manufacturer’s protocol. This DNA was used for the quantification of bacterial loads.

On day 10 after emergence, five GD and CV workers from each cage were sampled for gene expression analysis. The sampled legs, antennas, midguts and hindguts were then pooled for RNA extraction using RNA pure Total RNA Kit (Aidlab Biotechnologies Co. Ltd., Beijing, China) according to the manufacturer’s protocol. The cDNA synthesis reaction was performed using 0.5 μg total RNA with PrimeScript™ RT Master Mix (Takara Biomedical TechnologyCo., Ltd).

Ten AT and NF workers from each cage were sampled on day five after antibiotic treatment. The whole gut of five workers was dissected and pooled together for RNA extraction and cDNA synthesis, then used for the quantification of 16s rRNA transcript abundance. The midgut and hindgut of the other five workers were dissected and pooled together for RNA extraction and cDNA synthesis, and these cDNA were used for gene expression analysis.

### Quantification of bacterial loads in the gut of honey bees

Bacterial loads of GD, CV, AT and NF workers were determined by qPCR using universal bacterial 16S rRNA primers as listed in Table 1. The 16s rRNA copy numbers and transcript abundance were quantified using the StepOne Plus real‐time PCR system and the thermal cycling condition was as follow: initial denaturing step of 95 °C for 30s, 40 amplification cycles of95 °C for 5s and 60 °C annealing for 30s, and melt curve analysis from 60 to 95°C at 0.5°C/5s increments to confirm expected dissociation curves. qPCR reaction mixtures were setup with 1 μl DNA or cDNA, 0.2 μl of forward and reverse primers (10 μM), 5 μL TB Green™ *Premix Ex Taq* (Takara Biomedical Technology Co., Ltd) and 3.6 μl distilled water.

**Table 1:** Primers used in our study.

Target gene name and accession No.		Sequence (5′–3′)	Amplicon size	Annealing temperature	Efficiency	References
CYP6AS1 (NM_001365200.1)	F:	GCGACCAATGCGAATGAAAC	144	60°C	97%	De Smet *et al.* (2017)
	R:	TCACGGCATTCCACCATTTC				
CYP6AS3 (XM_026444747.1)	F:	TCGAAAGGGACGAGGATATG	129	60°C	99%	De Smet *et al.* (2017)
	R:	AGTCATGGGATGCCTACTGG				
CYP6AS4 (XM_395671.6)	F:	GGCTGGATTTGAAACGTCAT	109	60°C	104%	De Smet *et al.* (2017)
	R:	CGCGTGGAATTCTTTCATTT				
CYP6AS10 (XM_016915831.2)	F:	TGGCAGTGTATCATTTTACAAAACA	196	60°C	105%	De Smet *et al.* (2017)
	R:	TGGTATTGGCTTGGGTCCAG				
CYP9Q1 (XM_006562301.3)	F:	TCGAGAAGTTTTTCCACCG	116	60°C	93%	Mao *et al.* (2011)
	R:	CTCTTTCCTCCTCGATTG				
CYP9Q2 (XM_392000.7)	F:	GATTATCGCCTATTATTACTG	127	60°C	101%	Mao *et al.* (2011)
	R:	GTTCTCCTTCCCTCTGAT				
CYP9Q3 (XM_006562300.3)	F:	GTTCCGGGAAAATGACTAC	107	60°C	92%	Mao *et al.* (2011)
	R:	GGTCAAAATGGTGGTGAC				
Arp1 (NM_001185146.1)	F:	ATGCCAACACTGTCCTTTCTGG	151	60°C	94%	Johnson (2015)
	R:	GACCCACCAATCCATACGGA				
Universal bacterial 16s rRNA	F:	AGAGTTTGATCCTGGCTCAG	328	60°C	110%	Powell *et al.* (2014)
	R:	CTGCTGCCTCCCGTAGGAGT				

### Profiling the gene expression of Cytochrome P450 enzymes

We assayed the transcript levels of the following genes: *CYP6AS1*, *CYP6AS3*, *CYP6AS4*, *CYP6AS10*, *CYP9Q1*, *CYP9Q2* and *CYP9Q3*, with housekeeping gene *Arp1* chosen as the reference control. Primers used were listed in Table 1. RNA extraction, cDNA synthesis and qPCR analysis were conducted as described above. The relative expression levels of the selected genes were quantified using the StepOne Plus real‐time PCR system and the thermal cycling condition was as described above. The relative expression level (RE) of target genes was calculated using the 2^−ΔΔCt^ method (Livak and Schmittgen, 2001).

### Exposure of honey bees to insecticides

Thiacloprid and fluvalinate were purchased from Sigma‐Aldrich (St. Louis, MO, USA). Pesticide stock solutions dissolved in DMSO were diluted in sugar syrup and fed *ad libitum* to workers in the following concentrations: thiacloprid high dosage: 35 mg l^−1^, thiacloprid low dosage: 17.5 mg l^−1^, fluvalinate high dosage: 400 mg l^−1^ and fluvalinate low dosage: 200 mg l^−1^. A specific demonstration of experimental design was shown in Table 2.

**Table 2:** Experimental design of pesticide exposure treatments.

Experiment	Group name	Description
CV vs. GD	CVC	conventional gut community workers treated with DMSO
	CVT	conventional gut community workers treated with pesticide
	GDC	gut microbiota deficient workers treated with DMSO
	GDT	gut microbiota deficient workers treated with pesticide
AT vs. NF	NFC	normally fed workers treated with DMSO
	NFT	normally fed workers treated with pesticide
	ATC	antibiotic treated workers treated with DMSO
	ATL	antibiotic treated workers treated with low dosage pesticide
	ATH	antibiotic treated workers treated with high dosage pesticide
PBO treatment	CV	conventional gut community workers
	CV + PBO	conventional gut community workers treated with PBO
	CV + THI	conventional gut community workers treated with thiacloprid
	CV + FLU	conventional gut community workers treated with fluvalinate
	CV + PBO+THI	conventional gut community workers treated with both PBO and thiacloprid
	CV + PBO+FLU	conventional gut community workers treated with both PBO and fluvalinate

GD and CV workers were divided into different groups on day six after emergence: GD workers treated with pesticide (GDT), GD control workers (GDC), CV pesticide treated‐workers (CVT) and CV control workers (CVC). Treated bees received sugar syrup *ad libitum* containing thiacloprid high dosage or fluvalinate high dosage, control bees received sugar syrup *ad libitum* containing the same concentration of the solvent (DMSO).

For PBO co‐treatment experiment, CV workers were randomly divided into six groups (40 workers/cage). Workers were treated with DMSO, PBO only (0.1% in syrup), high dosage thiacloprid only, high dosage fluvalinate only, PBO + high dosage thiacloprid or PBO + high dosage fluvalinate.

AT and NF workers were divided into different groups on day 11 after emergence: AT workers treated with high dosage pesticide (ATH), AT workers treated with low dosage pesticide (ATL), AT control workers (ATC), NF workers treated with high dosage pesticide (NFT) and NF control workers (NFC). Treated bees received sugar syrup *ad libitum* containing thiacloprid or fluvalinate, control bees received sugar syrup *ad libitum* containing the same concentration of the solvent (DMSO).

### Analysis of pesticide residue in honey bees

GD and CV workers treated with thiacloprid were sampled on day four post‐treatment, while GD and CV workers treated with fluvalinate were sampled on day five post‐treatment. AT and NF workers treated with thiacloprid were sample on day five post‐treatment, while AT and NF workers treated with fluvalinate were sampled on day seven post‐treatment. Thirty workers from each cage were collected and used for chromatographic analysis. To eliminate the possible effects of intestinal contents, the abdomen was removed and only the worker head as well as thorax were used. Worker samples were extracted with 4 ml methanol and purified with 0.45 μm filters.

Chromatographic analysis was conducted using Agilent 1200 Series (Agilent Technologies, Inc., Snata Clara, CA, USA) equipment, using a Sepax HP‐C18 column (150 × 4.6 mm, 5 μm; Sepax Technologies, Inc., Newark, DE, USA) and maintained at 30°C during analysis. The mobile phase consisted of aqueous phase A, water and organic phase B, methanol at a ratio of 63:37 (A:B) for thiacloprid analysis and a aqueous phase A, water and organic phase B, acetonitrile at a ratio of 10:90 (A:B) for fluvalinate analysis. The flow rate was 1 ml min^−1^ for both analyses. 5 μl of each sample was injected through an automatic sampler system and monitored at 242 nm (thiacloprid) or 254nm (fluvalinate). Analytic standards for thiacloprid and fluvalinate were purchased from Sigma‐Aldrich, standard external method was used for quantification, the LOQ of thiacloprid and fluvalinate are 0.06 and 0.10 ng ml^−1^, respectively.

### Exposure of bee gut bacteria to pesticides

To evaluate the possible direct detoxification ability of gut bacteria, hindguts from 6 CV workers and 6 GD workers were dissected and homogenized. Bacteria were enriched according to Ellegaard and Engel (2019), in brief, the hindgut was collected in bead‐beating tubes with 1 ml PBS and homogenized with a bead‐beater using glass‐beads (0.75–1 mm) for 30 s at speed 6.0, then homogenates were centrifuged at 600 *g* for 5 min and the supernatant was collected into new Eppendorf tubes. The samples were then centrifuged at 10 000 *g* for 10 min, the supernatant was removed and the bacterial pellets were re‐suspended in PBS. The suspension was again centrifuged at 600 *g* for 5 min and 10 000 *g* for 10 min. Then, the pelleted bacterial cells were cultured in tryptic soy broth (Raymann *et al.*, 2018b) with 100 μg ml^−1^ thiacloprid and 100 μg ml^−1^ fluvalinate for 48 h at 35℃ and 5% CO_2_. Then, supernatants of bacterial cultures were dried and re‐suspended in methanol (0.5 ml). The chromatographic analysis was conducted as described above.

### Data analysis

Statistical analysis was carried out using SPSS software version 22.0. For the P450 gene expression profiling and *in vitro* pesticide metabolism, statistical significance was calculated using independent sample *t*‐test. The Kaplan–Meier survival curve and log‐rank test were used for the survival analysis. The pesticide residues were compared using one‐way ANOVA following LSD method or Dunnett’s T3 method. All figures were generated in GraphPad Prism 7 (GraphPad Software, San Diego, CA, USA).

## Conflict of interest

None declared.

## Supporting information


**Fig. S1.** Bacterial colonization levels in the guts of workers. The left part of panel shows the total bacterial loads in the gut of gut microbiota deficient (GD) worker (n = 30) and conventional gut community (CV) workers (n = 30). The right part of panel shows the transcript abundance of bacterial 16S rDNA of the gut bacterial loads of antibiotic treated (AT) workers (n = 6) and normally fed (NF) workers (n = 6). *P < 0.05, independent *t*‐test.
**Fig. S2.** *In vitro* exposure of bee gut homogenate to thiacloprid and fluvalinate (n = 6 workers).
**Fig. S3.** The expression changes of P450 legs and antennas of GD workers and CV workers (n = 3). Error bars represent SD fold changes. “*” represents significant difference (*P* < 0.05, independent sample *t*‐test).
