# Supplementary material for: Honey bee (Apis mellifera) gut microbiota promotes host endogenous detoxification capability via regulation of P450 gene expression in the digestive tract
Source: Microb Biotechnol. 2020 Apr 27;13(4):1201–12. doi: 10.1111/1751-7915.13579 (PMC7264748; doi:10.1111/1751-7915.13579)
Supplement: Supplementary file 1 — Fig. S1. Bacterial colonization levels in the guts of workers. The left part of panel shows the total bacterial loads in the gut of gut microbiota deficient (GD) worker (n = 30) and conventional gut community (CV) workers (n = 30). The right part of panel shows the transcript abundance of bacterial 16S rDNA of the gut bacterial loads of antibiotic treated (AT) workers (n = 6) and normally fed (NF) workers (n = 6). *P < 0.05, independent t‐test. Fig. S2. In vitro exposure of bee gut homogenate to thiacloprid and fluvalinate (n = 6 workers). Fig. S3. The expression changes of P450 legs and antennas of GD workers and CV workers (n = 3). Error bars represent SD fold changes. “*” represents significant difference (P < 0.05, independent sample t‐test). [file MBT2-13-1201-s001.docx]

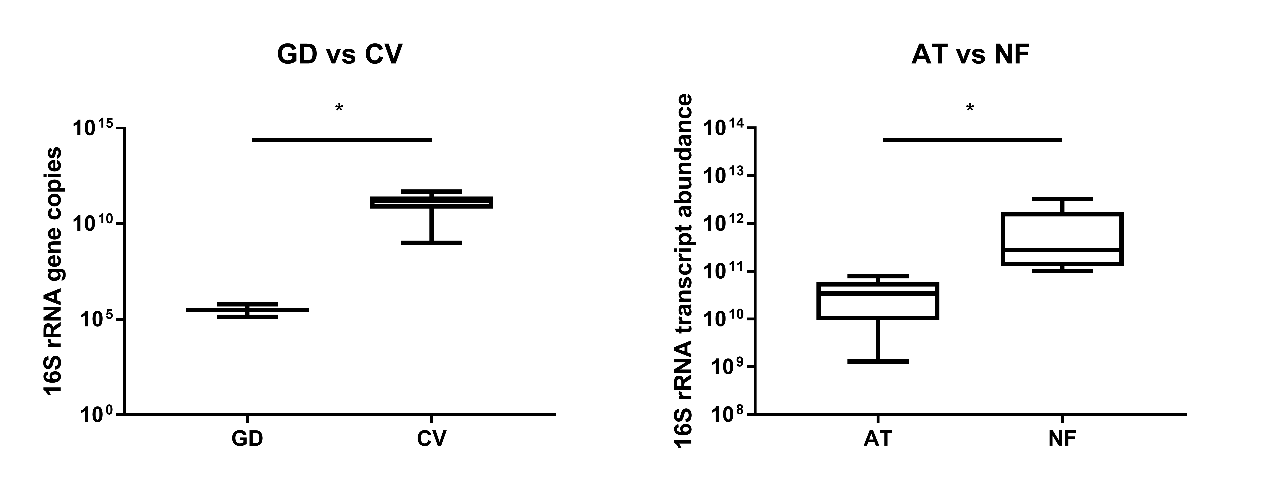


Figure S1. Bacterial colonization levels in the guts of workers. The left part of panel shows the total bacterial loads in the gut of gut microbiota deficient (GD) worker (n = 30) and conventional gut community (CV) workers (n = 30). The right part of panel shows the transcript abundance of bacterial 16S rDNA of the gut bacterial loads of antibiotic treated (AT) workers (n = 6) and normally fed (NF) workers (n = 6). **P* < 0.05, independent *t*-test.


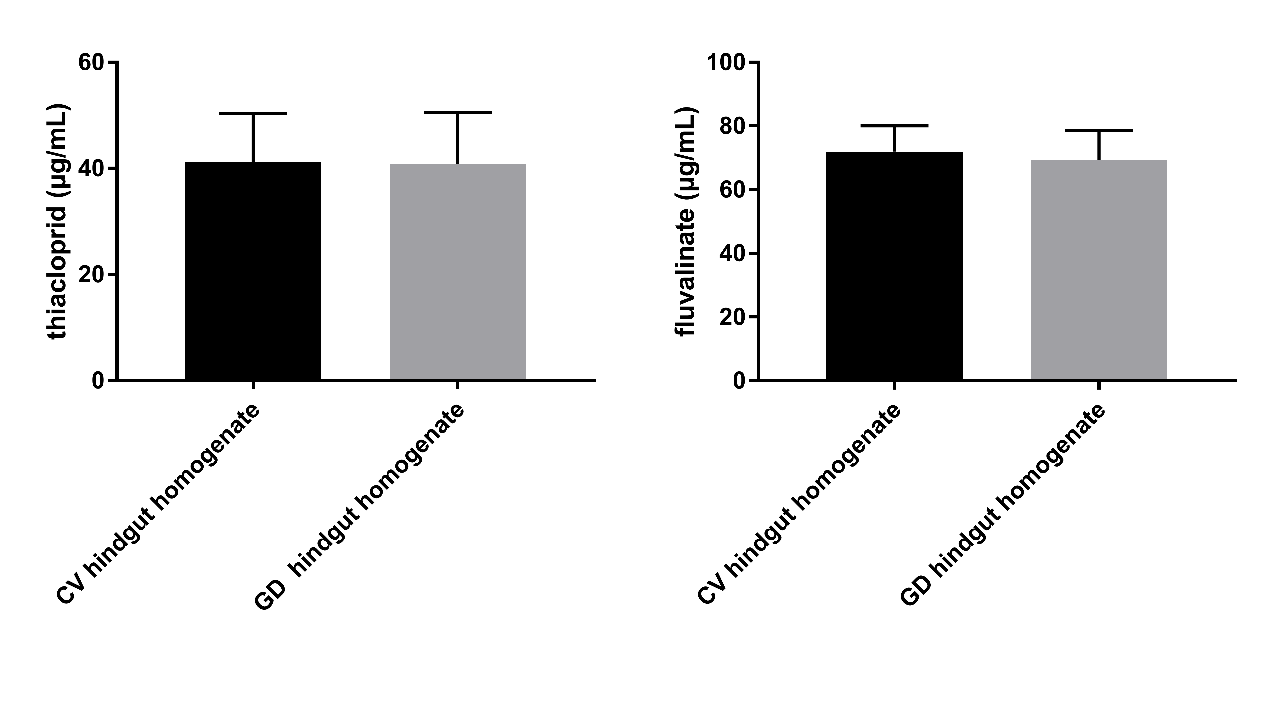


Figure S2. *In vitro* exposure of bee gut homogenate to thiacloprid and fluvalinate (n = 6 workers)


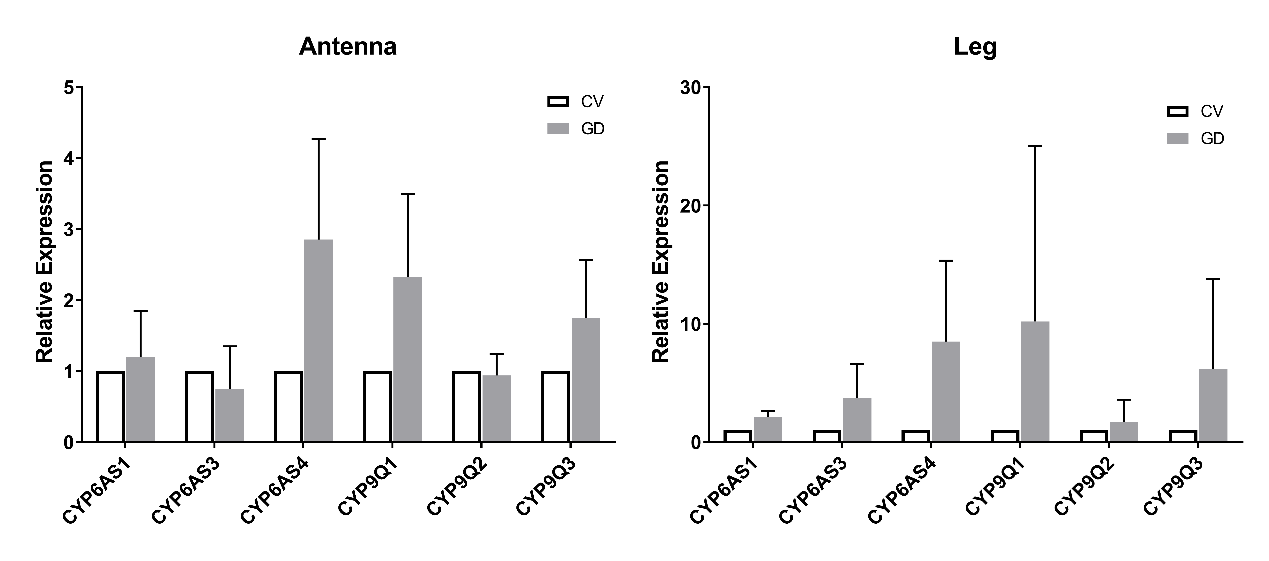


Figure S3. The expression changes of P450 legs and antennas of GD workers and CV workers (n = 3). Error bars represent SD fold changes. “*” represents significant difference (*P* < 0.05, independent sample t-test).
